# Tomography-based definition of keratoconus for Down syndrome patients

**DOI:** 10.1186/s40662-020-00215-1

**Published:** 2020-10-05

**Authors:** Soheila Asgari, Shiva Mehravaran, Mohammadreza Aghamirsalim, Hassan Hashemi

**Affiliations:** 1 Noor Ophthalmology Research Center, Noor Eye Hospital, No. 96 Esfandiar Blvd., Vali’asr Ave., Tehran, Iran; 2 ASCEND Center for Biomedical Research, Morgan State University, Baltimore, MD USA; 3 Translational Ophthalmology Research Center, Tehran University of Medical Science, Tehran, Iran

**Keywords:** Down syndrome, Keratoconus, Diagnostic criteria, Tomography, Discriminant analysis

## Abstract

**Background:**

To assess the diagnostic ability of Pentacam HR (Oculus Optikgeräte, GmbH, Wetzlar, Germany) tomographic indices in discriminating keratoconus (KC) and KC suspect (KCS) in 10- to 30-year-old patients with Down syndrome (DS).

**Methods:**

In this study, DS patients were enrolled through special needs schools, the National Down Syndrome Society, and relevant non-profit organizations. Diagnoses were made independently by two experienced specialists. Forty Pentacam indices related to corneal thickness, volume, density, keratometry, power, shape, aberration, and elevation were extracted. For each index, the accuracy for KC and KCS diagnosis was evaluated using discriminant analysis and the area under receiver operating characteristic curve (AUROC). From each enrolled case, data from only one eye was entered in the analyses.

**Results:**

Analyses were performed on data from 25 KC, 46 KCS, and 154 non-ectatic DS eyes. The best discriminants for KC were anterior higher order aberrations (HOA) (cutoff > 0.643, AUROC = 0.879), posterior vertical coma (cutoff > 0.0702 μm, AUROC = 0.875), anterior vertical coma (cutoff > 0.4124 μm, AUROC = 0.868), and total HOA (cutoff > 0.608, AUROC = 0.867). The difference between AUROCs were not statistically significant (all *P* > 0.05). For KCS, the best discriminants were minimum corneal thickness (cutoff ≤ 480.0 μm, AUROC = 0.775), corneal volume (cutoff ≤ 55.3 μm, AUROC = 0.727) and Belin Ambrosio display-total deviation (BAD-D) (cutoff > 2.23, AUROC = 0.718) with no significant difference between AUROCs (all *P* > 0.05).

**Conclusions:**

In this sample of DS patients, best KC discriminators were HOA and coma which showed good diagnostic ability. For KCS, best predictors were minimum corneal thickness, corneal volume, and BAD-D with relatively good diagnostic ability. Defining a new set of KC diagnostic criteria for DS patients is suggested.

## Background

Keratoconus (KC) is a degenerative inflammatory corneal pathology [1] that is usually associated with progressive thinning and steepening of the cornea [2]. Advanced techniques and 3-D assessment of the cornea have significantly enhanced our ability to detect KC and even KC suspects (KCS). Corneal topography is currently an important diagnostic tool, and its combination with pachymetry and assessment of corneal stiffness has improved diagnostic accuracy [3–5]. However, the diagnosis of KCS remains complicated and uncertain. There are several KC-related indices and classifications, especially for identifying cases in the early stages, but it can be a challenge for clinicians to consider them all. To facilitate the process, artificial intelligence methods (e.g., computer-aided diagnosis and artificial neural networks) have been developed, but the diagnosis of KC and KCS remains challenging [6].

The cornea in Down syndrome (DS) has a different structure, and DS patients have thinner and steeper corneas [7] compared to non-DS normal individuals. On the other hand, the prevalence of refractive errors in DS can be as high as 76.2% [8]. Therefore, the diagnosis of KC in this population is not only more challenging, but also important as it has implications for refractive surgery screening. The reported prevalence of KC in DS ranges from 0% [9] to 21.1% [10]. This difference, in addition to age and genetic predisposition, can be due to diagnostic criteria. Given these uncertainties in the diagnosis, the term KC-compatible has been used to describe findings, and it has been shown that 71.3% of DS patients may have findings suggestive of KC [11]. Given the lack of literature on this topic, this study was designed in an attempt to define diagnostic criteria for differentiating KC and KCS from non-ectatic corneas in DS patients.

## Material and methods

This research was done using data from a study of 10- to 30-year-old DS patients at Noor Eye Hospital in Tehran, Iran. The methodology of the study has been described elsewhere [12]. In brief, 250 DS patients were recruited through special needs schools, the National Down Syndrome Society, and relevant non-profit organizations, and 225 of them were enrolled into DS-KC, DS-KCS, and DS comparison (DS-C) groups. Inclusion criteria were diagnosis of DS and a minimum age of 10 years. Exclusion criteria were any concomitant mental illnesses such as autism or Klinefelter syndrome. For a normal control (NC) group, 200 normal subjects (non-DS) were matched by age and gender from normal cases presenting for a vision check-up (113 cases) and refractive surgery candidates presenting for their first work-up session (87 cases). For this report, we excluded cases with a history of ocular surgery due to their possible impact on the measured corneal indices.

### Ethical considerations

Approval for this study was obtained from the Ethics Committee of Tehran University of Medical Sciences (ID: 1397.091). The study adhered to the tenets of the Helsinki Declaration at all stages. Prior to enrollment, the goals and methods of the study were explained to the normal groups and parents of DS patients and written consent was obtained. For all cases in the three DS groups, informed consent were obtained from their parents/guardians, and participants were asked for verbal assent before any procedure.

### Examinations

Enrolled participants had complete ophthalmic examinations including slit-lamp biomicroscopy (Haag-Streit, Koniz, Switzerland), visual acuity testing using the SC-2000 Chart (Nidek co., Tokyo, Japan), retinoscopy using HEINE BETA 200 with ParaStop (HEINE Optotechnik, Herrsching, Germany), and imaging with Pentacam (Oculus Optikgeräte GmbH, Wetzlar, Germany). All Pentacam imaging was done by a skilled optometrist between 8 am and 12 noon. Imaging was repeated, if necessary, until an acceptable quality (minimum valid data: 93.0%) was acquired. If more than three attempts were needed, another appointment was scheduled for 2–3 days later to avoid participant fatigue and measurement error.

From the exported Pentacam data, the following 40 indices were used in this study:

1. Measures of corneal thickness, volume, and density: minimum corneal thickness (MCT), maximum Ambrósio’s relational thickness (ART-max), corneal volume, and total density in the 0–12 diameter;
2. Measures of keratometry and corneal shape: maximum keratometry in the central 3 mm (Ksteep), minimum keratometry in the central 3 mm (Kflat), maximum keratometry in the central 8 mm (Kmax), maximum keratometry in a 3 mm zone around the steepest point (ZKmax), anterior radius of curvature centered on the thinnest point (ARC), posterior radius of curvature centered on the thinnest point (PRC), index of surface variance (ISV), index of vertical asymmetry (IVA), keratoconus index (KI), center keratoconus index, index of height asymmetry, index of height decentration (IHD), inferior-superior asymmetry (I-S value), average of mean total corneal refractive power at 2 to 8 mm (mean TCRP), anterior asphericity, posterior asphericity, and total asphericity (Q-value);
3. Measures of corneal aberrations: anterior vertical coma, posterior vertical coma, anterior horizontal coma, posterior horizontal coma, anterior total coma, posterior total coma, total coma, anterior spherical aberrations (SA), posterior SA, total SA, anterior higher order aberrations (HOA), posterior HOA, and total HOA;
4. Measures of corneal elevation: anterior elevation at the thinnest point (AE-Thin), posterior elevation at the thinnest point (PE-Thin), anterior elevation at the apex (AE-Apex), and posterior elevation at the apex (PE-Apex);
5. The Belin Ambrosio display-total deviation (BAD-D).

### Definitions

Diagnoses of KC and KCS were made independently by two experienced specialists based on clinical and paraclinical criteria as described elsewhere [13]. Clinical criteria included Munson’s sign, Vogt’s striae, Fleischer’s ring, apical thinning, or Rizutti’s sign [14] and tomographic criteria were Kmax > 48.0 diopters (D) [15], ART-max < 339 μm [16], I-S value > 1.4 D [17], BAD-D > 1.6 [18], and posterior elevation. DS-KC cases were those who had the clinical signs and more than two abnormal tomography criteria. Patients in the DS-KCS group had no abnormal clinical finding and a maximum of two abnormal tomography criteria. Other cases were assigned to the DS-C group. From each case, data from only one eye was entered in the analyses. When fellow eyes met the criteria for the same group, the right eye was enrolled. In unilateral normal cases, the eye with KCS or KC was enrolled. For cases with KC in one eye and KCS in the other, the KC eye was enrolled.

### Statistical analysis

Statistical analyses were performed using SPSS version 21 (IBM Corp., Armonk, NY, USA) and MedCalc version 18.9.1 (MedCalc Software, Ostend, Belgium). To eliminate the effect of fellow eye correlations, data from only one eye per subject was used. First, variables that could discriminate DS-KC and DS-KCS from DS-C were identified using the leave-one-out cross-validation (LOOCV) technique. Discriminant analysis was used for selecting the best set of predictors, and accuracy was determined as the percentage of cases correctly classified. The discriminative ability of each parameter was then assessed using a receiver operating characteristic (ROC) curve. For each parameter, the area under curve (AUROC) was calculated, and the sensitivity and specificity values were determined based on the maximum Youden index. Finally, given the non-parametric distribution of AUROCs, we applied DeLong et al.’s method for making pairwise comparisons of AUROCs and comparing the performance of diagnostic tests. An AUROC of 0.90–1.00 represents excellent discrimination, 0.80–0.89 is considered good, 0.70–0.79 is fair, 0.60–0.69 is considered poor, and 0.50–0.59 is interpreted as very poor discrimination [19]. Cutoffs were determined by comparing values in the DS-KC and DS-KCS groups with those in the DS-C group. Values in the non-DS NC group were summarized to illustrate the differences between the non-ectatic DS-C group with an age-matched normal sample.

## Results

Data from 25 DS-KC eyes, 46 DS-KCS eyes, 154 DS-C right eyes, and 200 right eyes of the non-DS NC group were used. Compared to the DS-C group (16.73 ± 4.70 years), mean age was 16.56 ± 4.22 years in the DS-KC group (*P* = 0.852) and 18.06 ± 4.71 years in the DS-KCS group (*P* = 0.097). Although, compared to the DS-C group (55.2%), there were smaller proportions of females in the DS-KC (44.0%, *P* = 0.257) and DS-KCS groups (45.8%, *P* = 0.298), these differences were not significant.

Mean age (17.20 ± 4.36 years, *P* = 0.344) and proportion of females (49.5%, *P* = 0.394) in the NC group were not statistically significantly different from the DS-C group. One-way ANOVA between the NC and DS-C groups showed significant differences for all variables except cylindrical error. The background information of the four study groups is summarized in Table 1.

**Table 1:** Summary characteristics of eyes in the Down syndrome (DS) keratoconus (DS-KC), DS KC suspect (DS-KCS), DS non-ectatic comparison (DS-C), and non-DS normal control (NC) groups

Index	DS-KC (*n* = 25)	DS-KCS (*n* = 46)	DS-C (*n* = 154)	NC (*n* = 200)	*P* value*
UDVA (logMAR)	0.73 ± 0.57	0.58 ± 0.49	0.55 ± 0.44	0.91 ± 0.64	0.358
CDVA (logMAR)	0.29 ± 0.14	0.24 ± 0.13	0.23 ± 0.13	0.02 ± 0.08	0.279
Spherical error (D)	−1.51 ± 4.91	−0.40 ± 4.31	−0.21 ± 3.13	− 3.31 ± 3.38	0.428
Cylindrical error (D)	−2.54 ± 2.40	− 1.92 ± 1.21	−1.50 ± 1.06	−1.88 ± 1.53	0.009
Kmax (D)	50.26 ± 5.88	47.47 ± 1.65	47.09 ± 1.70	44.93 ± 1.55	< 0.001
BAD-D	2.98 ± 1.44	2.26 ± 0.66	1.72 ± 1.02	1.20 ± 0.53	< 0.001

*UDVA =* uncorrected distance visual acuity; *CDVA =* corrected distance visual acuity; *Kmax =* maximum keratometry at 8-mm zone; *BAD-D =* Belin Ambrosio display-total deviation

*One-way ANOVA between the three DS groups

### Keratoconus

**Fig. 1:**
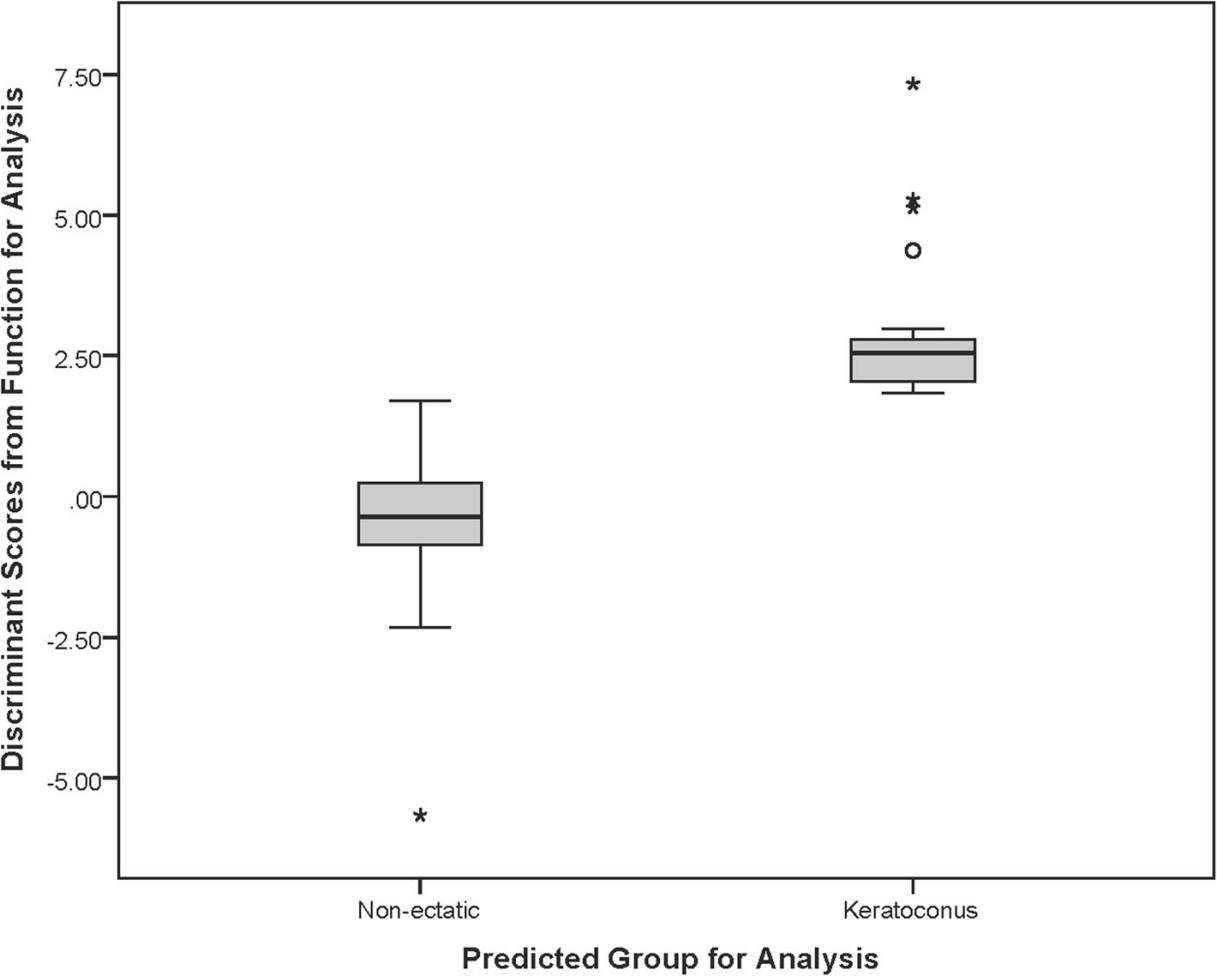
Distribution of discriminant scores in the keratoconus and non-ectatic groups of Down syndrome cases in this study

In discriminant analysis, differences between DS-KC and DS-C groups were statistically significant for all indices (all *P* < 0.05), except corneal volume, posterior asphericity, and anterior, posterior, and total SA. The differences of log determinants were non-significant (*P* < 0.001). The log determinants were relatively similar between DS-KC and DS-C groups, and although Box’s M rejected the assumption of equality of covariance matrices, the sample size made the test sufficiently robust. In discriminant function, the canonical correlation was 0.740, but structure matrix revealed 20 significant predictors: anterior vertical coma (0.609), total vertical coma (0.586), posterior vertical coma (0.567), IVA (0.526), anterior HOA (0.521), ISV (0.513), total HOA (0.512), KI (0.470), I-S value (0.437), IHD (0.425), ZKmax (0.410), Kmax (0.407), BAD-D (0.405), ARC (− 0.388), PRC (− 0.381), AE-Thin (0.366), anterior horizontal coma (0.359), total horizontal coma (0.356), PE-Thin (0.321), and Ksteep (0.310). All other indices showed weak correlations (≤ 0.3) with each discriminant function. The cross-validation table showed 95.30% correct classification (84.21% for DS-KC and 96.05 for DS-C). Figure 1 shows the distribution of discriminant scores in the DS-KC and DS-C groups.

Table 2 presents the AUROC and the sensitivity and specificity of these 20 indices in distinguishing DS-KC from DS-C cases. Based on AUROC classification, the best discriminators in descending order were the 11 indices of anterior HOA, posterior vertical coma, anterior vertical coma, total HOA, total vertical coma, I-S value, IVA, ISV, IHD, KI, and BAD-D. Among these good discriminators, the AUROC differences between anterior HOA, total HOA, anterior vertical coma, and posterior vertical coma were not statistically significant (all *P* > 0.05). ARC, PRC, ZKmax, Kmax, anterior horizontal coma, total horizontal coma, and Ksteep were fair discriminators. Among fair discriminators, only AUROC differences between Ksteep and ARC (*P* = 0.0095), ZKmax (*P* = 0.0016), and Kmax (*P* = 0.0079) were statistically significant.

**Table 2:** Results of discriminant analysis in differentiating between DS-KC (*n* = 46 eyes) and DS-C (*n* = 154 eyes) groups

Index	AUROC	Cutoff	Sensitivity (%)	Specificity (%)	Correct classification (%)
Anterior HOA	0.879	> 0.643	86.96	82.24	82.86
Posterior vertical coma	0.875	> 0.0702	91.30	76.97	78.86
Anterior vertical coma	0.868	> 0.4124	73.91	93.42	90.86
Total HOA	0.867	> 0.608	95.65	67.76	71.43
Total vertical coma	0.852	> 0.3665	73.91	89.47	87.43
I-S value	0.851	> 1.13	84.00	83.77	83.80
IVA	0.846	> 0.26	80.00	79.22	79.33
ISV	0.821	> 39.0	56.00	91.56	86.59
IHD	0.807	> 0.027	56.00	91.56	86.59
KI	0.806	> 1.05	84.00	64.94	67.60
BAD-D	0.802	> 2.45	70.83	84.21	82.39
ARC	0.768	≤ 7.19	64.00	83.12	80.45
PRC	0.754	≤ 5.91	68.00	79.22	77.65
ZKmax	0.751	> 47.34	68.00	75.16	74.16
Kmax	0.741	> 49.91	48.00	94.81	88.27
Anterior horizontal coma	0.722	> 0.3102	78.26	59.87	61.45
Total horizontal coma	0.719	> 0.323	78.26	61.18	63.43
Ksteep	0.702	> 48.50	52.00	87.66	82.68
Anterior elevation at thinnest point	0.696	> 8.0	48.00	94.74	88.13
Posterior elevation at thinnest point	0.689	> 14.0	60.00	85.53	81.92

*HOA =* higher order aberrations; *I-S value =* inferior-superior asymmetry; *IVA =* index of vertical asymmetry; *ISV =* index of surface variance; *IHD =* index of height decentration; *KI =* keratoconus index; *BAD-D =* Belin Ambrosio display-total deviation; *ARC =* anterior radius of curvature centered on thinnest point; *PRC =* posterior radius of curvature centered on thinnest point; *ZKmax =* maximum keratometry in a 3 mm zone around the steepest point; *Kmax =* Maximum keratometry at 8 mm; *Ksteep =* maximum keratometry at central 3 mm

### Keratoconus suspect

**Fig. 2:**
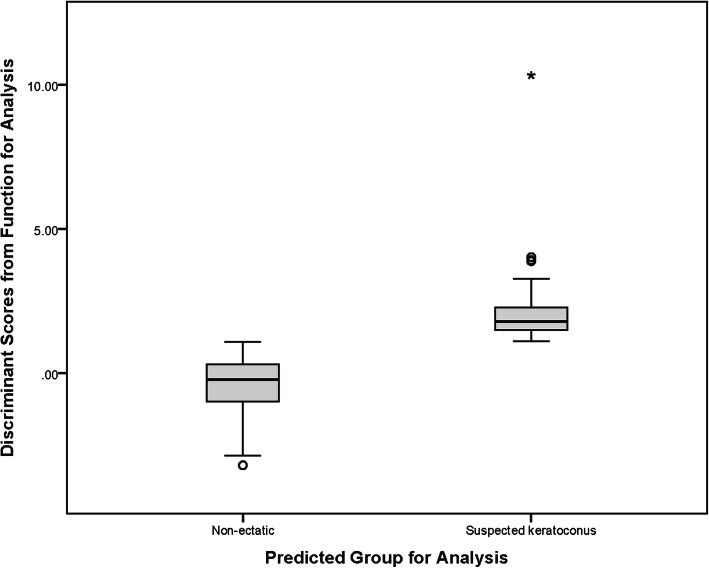
Distribution of discriminant scores in the keratoconus suspect and non-ectatic groups of Down syndrome cases in this study

In discriminant analysis, differences between DS-KCS and DS-C groups were statistically significant for Kflat, ARC, MCT, corneal volume, ART-max, IVA, KI, IHD, I-S value, BAD-D, AE-Thin, anterior vertical coma, posterior vertical coma, and total vertical coma (all *P* < 0.05). The log determinants were quite similar between DS-KCS and DS-C groups, and although Box’s M rejected the assumption of equality of covariance matrices, the sample size made the test sufficiently robust. Although the canonical correlation was 0.726, Wilks’ lambda showed significant discriminant function (*P* < 0.001). In the structure matrix, only the three indices of MCT (− 0.579), corneal volume (− 0.430), and BAD-D (0.318) were significant predictors. All other indices showed weak correlations (≤ 0.3) with each discriminant function. The cross-validation table showed 85.30% correct classification (78.79% for DS-KCS and 86.58% for DS-C). Figure 2 shows the distribution of discriminant scores for DS-C and DS-KCS groups.

Table 3 shows the AUROC and the sensitivity and specificity of these indices in distinguishing DS-KCS cases from DS-C. Based on AUROC classification, the best discriminators in descending order were the three indices of MCT, corneal volume, and BAD-D; all three demonstrated fair discrimination ability with no significant differences.

**Table 3:** Results of discriminant analysis in differentiating keratoconus suspect (*n* = 46 eyes) and non-ectatic corneas (*n* = 154 eyes) in Down syndrome patients

	AUROC	Cutoff	Sensitivity (%)	Specificity (%)	Correct classification (%)
MCT	0.775	≤ 480	66.67	90.91	85.15
Corneal volume	0.727	≤ 55.3	66.67	75.32	73.27
BAD-D	0.718	> 2.23	64.58	76.32	73.50

*MCT =* minimum corneal thickness; *BAD-D =* Belin Ambrosio display-total deviation

The mean values of the main discriminating indices in the four study groups are summarized in Table 4.

**Table 4:** Mean ± standard deviation of the main discriminators in the Down syndrome (DS) keratoconus (DS-KC), DS keratoconus suspect (DS-KCS), DS non-ectatic comparison (DS-C), and non-DS normal control (NC) groups of the study

Index	DS-KC (*n* = 25)	DS-KCS (*n* = 46)	DS-C (*n* = 154)	NC (*n* = 200)
Minimum corneal thickness (μm)	484.12 ± 32.34	492.28 ± 37.57	515.48 ± 30.36	546.9 ± 40.5
Corneal Volume (mm^3^)	57.45 ± 4.37	55.13 ± 3.35	57.62 ± 3.26	61.19 ± 4.04
Belin Ambrosio display-total deviation	2.98 ± 1.44	2.26 ± 0.66	1.72 ± 1.02	1.20 ± 0.53
Anterior HOA (μm)	1.13 ± 1.00	0.60 ± 0.17	0.55 ± 0.18	0.37 ± 0.09
Posterior vertical coma (μm)	0.15 ± 0.19	0.06 ± 0.04	0.05 ± 0.03	0.02 ± 0.02
Anterior vertical coma (μm)	0.70 ± 0.77	0.29 ± 0.19	0.20 ± 0.13	0.14 ± 0.09
Total HOA (μm)	1.12 ± 0.89	0.62 ± 0.17	0.57 ± 0.19	0.38 ± 0.12

Differences between the four groups were significant by one-way ANOVA and post hoc Scheffe for all indices except aberration indices between DS-KCS and DS-C groups

## Discussion

Several studies have reported variable rates for the prevalence and incidence of KC in DS patients, which may be due to the lack of standard criteria for KC diagnosis in DS [11]. As demonstrated in this study (Tables 1 and 4), the DS-C group differed from the age- and gender-matched non-DS NC group. Overall, the findings suggest that thickness variables can be useful in the diagnosis of KCS, while coma indices and dioptric asymmetry are better indicators for the diagnosis of KC in DS patients. Table 5 compares the suggested cutoff values and their sensitivity and specificity levels for anterior HOA, posterior vertical coma, anterior vertical coma, and total HOA in the diagnosis of KC and for MCT in the diagnosis of KCS in DS patients of the present study compared to non-DS young patients in other studies.

**Table 5:** Comparison of suggested cutoff values and their sensitivity and specificity levels for the diagnosis of keratoconus (KC) and KC suspect (KCS) in Down syndrome (DS) patients of the present study compared to non-DS young patients in other studies

		DS patients			Non-DS patients		
		Cutoff	Sensitivity (%)	Specificity (%)	Cutoff	Sensitivity (%)	Specificity (%)
KC	Anterior HOA	> 0.643	86.96	82.24	> 0.801 [20]	100.0	100.0
	Posterior vertical coma	> 0.0702	91.30	76.97	> 0.055 [20]	100.0	100.0
	Anterior vertical coma	> 0.4124	73.91	93.42	> 0.200 [21]	94.1	96.7
	Total HOA	> 0.608	95.65	67.76	> 0.908 [22]	86.5	81.1
KCS	Minimum corneal thickness	≤ 480.0	66.67	90.91	< 512.0 [23]	64.0	58.0
	Corneal volume	≤ 55.3	66.67	75.32	< 23.95 [24]	79.0	79.3
	BAD-D	> 2.23	64.58	76.32	> 1.6 [25]	83.8	86.0

*HOA =* higher order aberrations; *BAD-D =* Belin Ambrosio display-total deviation

In terms of corneal thickness, two studies [7, 26] have shown thicker corneas in DS patients compared to the non-DS population. In contrast, Aslan et al. [10] reported thinner corneas and suggested that thickness measures could increase the diagnostic accuracy for early KC in DS patients. In our study [27], corneal thickness was assessed from the center to the periphery with Pentacam, and thickness values in DS-C patients were lower in all corneal segments compared to the non-DS NC group. Examining the diagnostic ability of corneal thickness indices in non-DS normal subjects, Muftuoglu et al. [23] arrived at results similar to ours and found thickness indices to be fair discriminators for forme frust KC (FFKC). The cutoff values they suggested for MCT was 512 μm for detecting FFKC with a specificity level of 58%. For the DS sample in our study, although the AUROC differences between MCT, corneal volume, and BAD-D were not significant, MCT had the best specificity (90.91%) and accuracy (85.15%) for differentiating DS-KCS from DS-C with a cutoff of 480 μm. However, thickness measures showed weaker diagnostic ability for DS-KC.

Keratometric indices have been suggested to have good and excellent diagnostic power for distinguishing KC [28] and KCS [23] from normal corneas in non-DS populations. In this study, however, these indices were poor and very poor in detecting KCS in DS patients. In the sample population of our study, central-peripheral keratometric indices indicated steeper corneas in DS-C cases compared to the NC group [29]. This difference significantly reduces the diagnostic power of these indices, especially Kmax, for milder cases. But for DS-KC, they perform as fair discriminators. In this set of indices, dioptric asymmetry indices detected KC better than corneal slope. As such, IVA, I-S value, IHD, and ISV had similar or better discriminating power than K values.

In our study, among aberrations, anterior HOA, total HOA, anterior vertical coma, and posterior vertical coma had the highest diagnostic power in the diagnosis of KC and were all similarly good discriminators. In a non-DS population, Saad and Gatinel [30] showed that the total coma measured by the OPD Scan is a fair discriminator of FFKC and an excellent discriminator for KC. Hashemi et al. [31] also reported 3rd order vertical coma to be a fair discriminator for the diagnosis of KCS in non-DS subjects, and an excellent discriminator for clinical KC. While coma can serve as a diagnostic parameter for KC in DS patients, like other indices, its diagnostic power is lower in DS patients compared to non-DS subjects.

In non-DS samples, elevation indices appear to have higher sensitivity and specificity in detecting early stages of KC compared to other indices measured by Pentacam [32], and they are suggested to have excellent discrimination power for KC [28, 33, 34]. However, in our sample of DS patients, elevation indices had poor diagnostic power; at best, AE-Thin was a poor discriminator for KCS and AE-Apex was a fair discriminator for KC. Therefore, elevation indices appear to be less predictive in DS patients.

One of the limitations of the present study was the small sample size in the KC group, which is due to the limited number of DS patients for the total sample of the study. As a result, we were not able to perform subgroup analyses based on KC severity. Also, due to this limitation, we could not use machine learning approaches which can be better tools for KC diagnosis and staging [35]. Furthermore, availability of non-tomographic indices such as corneal biomechanics might have enabled us to increase the diagnostic accuracy for KC. Despite these limitations, to our knowledge, this is the first study with a large sample (*n* = 225) of 10- to 30-year-old DS patients to establish diagnostic criteria for the diagnosis of KC.

## Conclusions

In conclusion, the cutoffs calculated in this study suggest that a new set of diagnostic criteria for keratoconus need to be defined for DS patients (Table 5). In this sample of DS patients, best KC discriminators were HOA and coma which showed good diagnostic ability. For KCS, best predictors were MCT, corneal volume, and BAD-D with relatively good diagnostic ability. Customized criteria can help with the early identification and treatment of KC, especially for those undergoing screening for refractive surgery.

## Data Availability

The data will be available in the case of reasonable request by the corresponding author.

## Abbreviations

DS-KC: Down syndrome keratoconus
DS-KCS: Down syndrome keratoconus suspect
DS-C: Down syndrome comparison
Non-DS NC: Non-Down syndrome normal control
ZKmax: Maximum keratometry in a 3 mm zone around the steepest point
Kmax: Maximum keratometry at 8 mm
Ksteep: Maximum keratometry at central 3 mm
Kflat: Minimum keratometry
I-S value: Inferior-superior asymmetry
IVA: Index of vertical asymmetry
ISV: Index of surface variance
IHD: Index of height decentration
IHA: Index of height asymmetry
CKI: Center keratoconus index
KI: Keratoconus index
ARC: Anterior radius of curvature centered on thinnest point
PRC: Posterior radius of curvature centered on thinnest point
TCRP: Total corneal refractive power
MCT: Minimum corneal thickness
ART-max: Maximum *Ambrósio* relational thickness
HOA: Higher order aberration
SA: Spherical aberrations
FFKC: Forme frust keratoconus
BAD-D: *Belin Ambrósio* Enhanced Ectasia *Display*-total deviation
AE-Thin: Anterior elevation at the thinnest point
PE-Thin: Posterior elevation at the thinnest point
AE-Apex: Anterior elevation at the apex
PE-Apex: Posterior elevation at the apex
LOOCV: Leave-one-out cross-validation
AUROC: Area under receiver operating characteristic curve
UDVA: Uncorrected distance visual acuity
CDVA: Corrected distance visual acuity

## References

[1] Hedayatfar A, Hashemi H, Aghaei H, Ashraf N, Asgari S (2019). Subclinical inflammatory response: accelerated versus standard corneal cross-linking. Ocul Immunol Inflam.

[2] Krachmer JH, Feder RS, Belin MW (1984). Keratoconus and related noninflammatory corneal thinning disorders. Surv Ophthalmol.

[3] Alió JL, Piñero DP, Alesón A, Teus MA, Barraquer RI, Murta J (2011). Keratoconus-integrated characterization considering anterior corneal aberrations, internal astigmatism, and corneal biomechanics. J Cataract Refract Surg.

[4] Ambrosio R, Lopes B, Faria-Correia F, Vinciguerra R, Vinciguerra P, Elsheikh A (2016). Ectasia detection by the assessment of corneal biomechanics. Cornea.

[5] Ambrosio R, Lopes BT, Faria-Correia F, Salomão MQ, Bühren J, Roberts CJ (2017). Integration of Scheimpflug-based corneal tomography and biomechanical assessments for enhancing ectasia detection. J Refract Surg.

[6] Alio JL, Vega-Estrada A, Sanz-Díez P, Peña-García P, Durán-García ML, Maldonado M (2015). Keratoconus management guidelines. Int J Kerat Ect Cor Dis.

[7] Aslan L, Aslankurt M, Aksoy A, Gumusalan Y (2014). Differences of the anterior segment parameters in children with Down syndrome. Ophthalmic Genet.

[8] Adio AO, Wajuihian SO (2012). Ophthalmic manifestations of children with Down syndrome in Port Harcourt, Nigeria. Clin Ophthalmol.

[9] Fimiani F, Iovine A, Carelli R, Pansini M, Sebastio G, Magli A (2007). Incidence of ocular pathologies in Italian children with Down syndrome. Eur J Ophthalmol.

[10] Aslan L, Aslankurt M, Yüksel E, Özdemir M, Aksakal E, Gümüşalan Y (2013). Corneal thickness measured by Scheimpflug imaging in children with Down syndrome. J AAPOS.

[11] Alio JL, Vega-Estrada A, Sanz P, Osman AA, Kamal AM, Mamoon A (2018). Corneal morphologic characteristics in patients with Down syndrome. JAMA Ophthalmol.

[12] Asgari S, Hashemi H, Fotouhi A, Mehravaran S (2020). Anterior chamber dimensions, angles and pupil diameter in patients with Down syndrome: a comparative population-based study. Indian J Ophthalmol.

[13] Hashemi H, Miraftab M, Amanzadeh K, Seyedian MA, Vinciguerra R, Ambrósio R (2020). Keratoconus detection by novel indices in patients with Down syndrome: a cohort population-based study. Jpn J Ophthalmol.

[14] Rabinowitz YS (1998). Keratoconus. Surv Ophthalmol.

[15] Rabinowitz YS, Rasheed K (1999). KISA% index: a quantitative videokeratography algorithm embodying minimal topographic criteria for diagnosing keratoconus. J Cataract Refract Surg.

[16] Ambrosio R, Caiado AL, Guerra FP, Louzada R, Sinha RA, Luz A (2011). Novel pachymetric parameters based on corneal tomography for diagnosing keratoconus. J Refract Surg.

[17] Burns DM, Johnston FM, Frazer DG, Patterson C, Jackson AJ (2004). Keratoconus: an analysis of corneal asymmetry. Br J Ophthalmol.

[18] Villavicencio GF, Henriquez MA, Izquierdo L, Ambrosio R, Belin MW (2014). Independent population validation of the Belin/Ambrosio enhanced ectasia display: implications for keratoconus studies and screening. Int J Kerat Ect Cor Dis.

[19] Diamond GA (1992). What price perfection? Calibration and discrimination of clinical prediction models. J Clin Epidemiol.

[20] Heidari Z, Mohammadpour M, Hashemi H, Jafarzadehpur E, Moghaddasi A, Yaseri M (2020). Early diagnosis of subclinical keratoconus by wavefront parameters using Scheimpflug, Placido and Hartmann-shack based devices. Int Ophthalmol.

[21] Bühren J, Kook D, Yoon G, Kohnen T (2010). Detection of subclinical keratoconus by using corneal anterior and posterior surface aberrations and thickness spatial profiles. Invest Ophthalmol Vis Sci.

[22] Naderan M, Jahanrad A, Farjadnia M (2018). Ocular, corneal, and internal aberrations in eyes with keratoconus, forme fruste keratoconus, and healthy eyes. Int Ophthalmol.

[23] Muftuoglu O, Ayar O, Ozulken K, Ozyol E, Akinci A (2013). Posterior corneal elevation and back difference corneal elevation in diagnosing forme fruste keratoconus in the fellow eyes of unilateral keratoconus patients. J Cataract Refract Surg.

[24] Cui J, Zhang X, Hu Q, Zhou WY, Yang F (2016). Evaluation of corneal thickness and volume parameters of subclinical keratoconus using a Pentacam Scheimflug system. Curr Eye Res.

[25] Shetty R, Rao H, Khamar P, Sainani K, Vunnava K, Jayadev C (2017). Keratoconus screening indices and their diagnostic ability to distinguish normal from ectatic corneas. Am J Ophthalmol.

[26] Karadag R, Erdurmus M, Yagci R, Keskin UC, Hepsen IF, Durmus M (2007). Central corneal thickness in individuals with intellectual disabilities. Cornea.

[27] Hashemi H, Makateb A, Mehravaran S, Fotouhi A, Shariati F, Asgari S (2020). Mapping the corneal thickness and volume in patients with Down syndrome: a comparative population-based study. Arq Bras Oftalmol.

[28] Yousefi A, Hashemi H, Khanlari M, Amanzadeh K, Aghamirsalim M, Asgari S (2020). Keratometric indices for detecting the type of keratoconus: a combined discriminant analysis. Clin Exp Optom.

[29] Asgari S, Mehravaran S, Fotouhi A, Makateb A, Hashemi H. Total corneal refractive power and shape in Down syndrome. Eur J Ophthalmol. 2019;1120672119883594. 10.1177/1120672119883594. 10.1177/1120672119883594 31635486

[30] Saad A, Gatinel D (2012). Evaluation of total and corneal wavefront high order aberrations for the detection of forme fruste keratoconus. Invest Ophthalmol Vis Sci.

[31] Hashemi H, Beiranvand A, Yekta A, Maleki A, Yazdani N, Khabazkhoob M (2016). Pentacam top indices for diagnosing subclinical and definite keratoconus. J Current Ophthalmol.

[32] Kamiya K, Ishii R, Shimizu K, Igarashi A (2014). Evaluation of corneal elevation, pachymetry and keratometry in keratoconic eyes with respect to the stage of Amsler-Krumeich classification. Br J Ophthalmol.

[33] Wahba SS, Roshdy MM, Elkitkat RS, Naguib KM (2016). Rotating Scheimpflug imaging indices in different grades of keratoconus. J Ophthalmol.

[34] Ucakhan OO, Cetinkor V, Ozkan M, Kanpolat A (2011). Evaluation of Scheimpflug imaging parameters in subclinical keratoconus, keratoconus, and normal eyes. J Cataract Refract Surg.

[35] Yousefi S, Yousefi E, Takahashi H, Hayashi T, Tampo H, Inoda S (2018). Keratoconus severity identification using unsupervised machine learning. PLoS One.

